# Dependence of Anterior Active Rhinomanometry Indices on Nasal Obstructive Disorders in Children with Atopic Bronchial Asthma Complicated by Nasal Symptoms

**DOI:** 10.1155/2018/1869613

**Published:** 2018-10-04

**Authors:** Tatyana I. Eliseeva, Svetlana V. Krasilnikova, Sergey Yu. Babaev, Alexey A. Novozhilov, Dmitry Yu. Ovsyannikov, Stanislav K. Ignatov, Nailya I. Kubysheva, Andrey V. Shakhov

**Affiliations:** ^1^MD, DSc, Professor, Chair of Hospital Pediatrics, Privolzhsky Research Medical University, 10/1 Minin and Pozharsky Square, Nizhny Novgorod 603005, Russia; ^2^Assistant, Department of ENT Diseases, Privolzhsky Research Medical University, 10/1 Minin and Pozharsky Square, Nizhny Novgorod 603005, Russia; ^3^MD, Department of ENT Diseases, Privolzhsky Research Medical University, 10/1 Minin and Pozharsky Square, Nizhny Novgorod 603005, Russia; ^4^MD, Head of the Department of ENT Diseases, Privolzhsky District Medical Center of Federal Medico-Biologic Agency of Russia, 2 Nizhne-Volzhskaya Naberezhnaya, Nizhny Novgorod 603005, Russia; ^5^MD, DSc, Head of the Department of Pediatrics, Medical Institute, Peoples' Friendship University of Russia (RUDN University), 6 Miklukho-Maklaya St., Moscow 117198, Russia; ^6^DSc, Professor, Chair of Photochemistry and Spectroscopy, Lobachevsky State University of Nizhny Novgorod, 23 Gagarin Avenue, Nizhny Novgorod 603950, Russia; ^7^DSc, Senior Researcher, Medical Informatics Research Laboratory of the Higher School of Information Technologies and Information Systems, Kazan Federal University, 18 Kremlyovskaya St., Kazan 420000, Russia; ^8^MD, DSc, Professor, Head of the Department of ENT Diseases, Privolzhsky Research Medical University, 10/1 Minin and Pozharsky Square, Nizhny Novgorod 603005, Russia

## Abstract

**Background:**

Atopic bronchial asthma (BA) in children is associated with upper airways pathology (UAP). Among them, a combination of allergic rhinitis (AR) and nasal obstructive disorders (NOD), including hypertrophy of the pharyngeal tonsil (HPT) and anomalies of the intranasal structures (AINS), is abundant. In such patients, anterior active rhinomanometry (AARM) is an important method of examining nasal patency. However, NOD can influence the AARM parameters in children with BA and nasal symptoms, and this effect must be taken into account in clinical practice.* Study goal* was to elucidate the effect of NOD on rhinomanometric parameters in this group of patients.

**Methods:**

Total of 66 children with BA and AR were examined with AARM, rhinovideoendoscopy, spirometry, and standard clinical tests allowing revealing the structure of comorbid pathologies. In order to avoid the influence of anthropometric parameters of children and their age on AARM parameters, a special index of reduced total nasal airflow was used.

**Results:**

It has been established that NOD, especially HPT, have a significant negative impact on the indices of anterior active rhinomanometry during the periods of both AR remission and AR exacerbation. The effect of AINS is much weaker and was remarkable only in combination with HPT.

## 1. Introduction

Bronchial asthma (BA) is a common chronic airway disease in children which, in most patients, is associated with allergic rhinitis (AR) [1–4]. AR as well as allergic rhinosinusitis may contribute to worsening asthma control and complicating diagnostic and therapeutic management of asthmatic patients, especially in severe asthma [5]. AR in asthmatics children often combines with other upper airway pathologies (UAP), in particular, with nasal obstructive disorders (NOD) which includes hypertrophy of the pharyngeal tonsil (HPT) and/or anomalies of intranasal structures (AINS) [3, 6, 7]. UAP have a negative effect on the course of asthma requiring timely, objective monitoring of the basic physiological functions involved in the pathological process. Such functions, in the first place, are bronchial and nasal respiratory patency [8–10].

The standard method for assessing bronchial patency is the method of spirometry, which is included in current recommendations for the management of patients with asthma [1, 11]. When examining nasal respiratory patency, the anterior active rhinomanometry (AARM) is frequently considered as a promising candidate for a “gold standard” of measurements. This method is highly sensitive and noninvasive and requires minimal cooperation with a patient and excludes his subjective assessments, which is especially important in pediatrics [12–14]. The widespread usage of AARM in the clinical practice is hampered, among other factors, by the lack of information on the dependence of the AARM results on the comorbid pathologies and variation of anthropometric characteristic of patients and their conditions. Probably, this explains the lack of this method in the list of recommendations for managing patients with AR [9, 15].

There are only few studies of nasal patency evaluation with the objective methods in asthmatic patients [9, 16]. They demonstrate the mutual influence of respiratory patency of the upper and lower airways [16]. Unfortunately, these studies do not take into account the possible impact on nasal respiratory patency of NOD, which, along with AR, occurs in a significant proportion of asthmatic children [2, 17]. These diseases, including nasal septum deformity, can exacerbate nasal obstruction caused by allergic inflammation of the mucosa in AR and cause insufficient response to ongoing anti-inflammatory therapy [18]. We have not found any studies where objective assessment of the effect of NOD on nasal flow parameters in children with asthma compared with patients without NOD would be made. In particular, in the study of Chen et al. [9], the AARM method was used to study nasal patency in children with asthma and nasal symptoms, but patients with anatomical deformations of upper airways were excluded from the study cohort. At the same time, according to our data, up to 50% of children with asthma and nasal symptoms have such deformations [2, 7]. Motomura et al. [19] demonstrated that, for patients with asthma, a decrease in nasal airflow is characteristic, but the effect of the comorbid UAP on the respiratory function of the nose was not considered. Yukselen A. et al. [20] established a significant relationship between spirometric parameters and nasal airflow in children with asthma. However, the effect of NOD on the respiratory function of the nose in this study was also not taken into account [20].

A discussion remains about the association of nasal symptoms and rhinomanometric indicators in patients with AR. In the studies of Ciprandi et al. [21] and Mozzanica et al. [22], a significant correlation between the clinical evaluation of AR symptoms and rhinomanometric parameters was demonstrated. At the same time, according to Keeler and Most [23], objective indices of nasal obstruction do not always correlate with subjective estimates of this syndrome [23]. Unexpected results in the study of children and adolescents with AR were obtained by Mendes et al. [24]. The authors did not find a correlation between objective and subjective measurements, when the nasal cavity was assessed as a whole, but a significant negative correlation was found between these parameters when each nostril was evaluated individually. Children with HPT and AINS (primarily deviations of the nasal septum) were not included in the study. Similar results were obtained in the study of Roithmann et al. [25], where no significant correlation was found between the subjective sensations of nasal patency and the general resistance of airflow.

Thus, the available results leave unclear the question on how accurately the results of AARM method determine the objective status of asthmatic patients with AR under conditions of comorbid pathologies including NOD. Timely objectification and precise diagnosis of the effect of these pathological conditions on the respiratory function of the nose would enable us to assess the real clinical picture and prospects of both conservative therapy and the need for surgical correction in children with torpid nasal symptoms due to the combination of AR and NOD [3, 6, 7, 26].

An additional complication in the interpretation of rhinomanometric parameters in children is the potential dependence of these parameters on the age and anthropometric parameters of a child. At present, there are studies that characterize rhinomanometric indicators in children in selected populations [27–29], but unified recommendations for evaluating AARM parameters in children have not been developed to date [13]. At the same time, in studies of Chen et al. [9] and Julia et al. [28], the relationship between the results of AARM and the anthropometric parameters of children was noted. According to [9], as the child grows, the size of the nasal cavity increases, which causes an increase in nasal airflow. However, it should be kept in mind that anthropometric data, including height, in children with asthma may have an association with the severity of asthma [30]. Obviously, there is an urgent need for the development of unified reference values of AARM for children, in analogy with the proper parameters available for spirometry, taking into account age, sex, race, anthropometric data, and the BA severity of patients.

Thus, the current lack of data on the NOD influence on the parameters of AARM in children with asthma and nasal symptoms is an objective factor that reduces the reliability and diagnostic significance of AARM in conditions of real clinical practice in monitoring nasal respiratory function. In this study, we investigate this issue by comparing the AARM parameters in children with asthma and nasal symptoms, taking into account the presence or absence of a combination of AR and NOD, as well as the period (remission or exacerbation) of AR in these patients. In addition, we pay special attention to the influence of the age of patients on the characteristics under study and introduce additional characteristics that allow account for the influence of the size of the nasal cavity on monitoring parameters.

## 2. Materials and Methods

### 2.1. Formation of the Cohort of Patients

The study was conducted according to the Helsinki Declaration adopted in June 1964 (Helsinki, Finland) and revised in October 2000 (Edinburgh, Scotland). The study was approved by the Ethics Committee of Privolzhsky Research Medical University, protocol No 13 of 10.10.16. Informed consent was obtained from the patients between 15 and 17 years old and from the parents of patients under the age of 15, according to the Federal Law “Fundamentals of the Legislation of the Russian Federation on the Protection of Health of Citizens” of July 22, 1993, No. 5487-1.

A total of 66 children and adolescents aged from 4 to 17 years were examined, the average age was 8.2±4.3, and boys amounted for 60.6% (40/66), who were on treatment for asthma at the Children's City Clinical Hospital No. 1 of Nizhny Novgorod, Russia, and who had nasal or sinonasal complaints. All the children had a symptomatic complex that was characteristic of BA and AR, family anamnesis associated with atopy was evaluated (asthma, AR, conjunctivitis, atopic dermatitis, and urticaria), positive skin test results were obtained, or high titers of specific class E immunoglobulins were detected at least for one of the most common aeroallergens of the Volga-Vyatka region of the Russian Federation.

Criteria for inclusion were the diagnosis of asthma, made in accordance with the existing international and national conciliation documents, the presence of nasal or sinonasal complaints and symptoms in patients [1]. Exclusion criteria presuppose the presence of acute infectious diseases and fever, diabetes, autoimmune disorders, primary immunodeficiencies, and cancer. Besides, children who had symptoms of hypertrophic rhinitis were not included in this study. Treatment of BA and comorbid UAP was carried out in accordance with the existing conciliation documents taking into account modern therapeutic strategies [1, 31].

Depending on the comorbid UAP pathologies, four groups of patients were considered: Group 1 (BA patients with isolated AR (without NOD)), Group 2 (BA patients with AR+HPT), Group 3 (BA patients with AR+AINS), and Group 4 (BA patients with a combination AR+HPT+AINS). In order to assess the effect of AR severity, two groups of patients were additionally considered in a whole cohort: Group A (patients with AR in the remission period) and Group B (patients with AR in the exacerbation period).

### 2.2. Objective and Subjective Measurements

Due to the presence of nasal symptoms, all the patients were examined by an otorhinolaryngologist, who conducted a routine examination and a rhino video-endoscopic examination of upper airways. Rigid rhinoscopes from the company Karl Storz (Germany) with a viewing angle of 0 and 30°, diameter of 2.7 and 4.0 mm, and a flexible nasopharyngoscope 3.2 mm were used. Video recording was done by means of Atmos camera (Germany). Rhinovideoscopic examination was performed after instillation of a 2% solution of lidocaine on the nasal mucosa and application of anesthesia using 0.1% solution of epinephrine hydrochloride and 10% lidocaine. AR and allergic rhinosinusitis were diagnosed in accordance with the available international recommendations. The involvement of sinuses in the pathological process was noted using the criteria of the European Position Paper on Rhinosinusitis and Nasal Polyps (EPOS), 2012 [32]. In assessing UAP, the diagnosis was verified using the International Classification of Diseases, 10th revision (ICD-X), the recommendations of the Allergic Rhinitis and its Impact on Asthma (ARIA) 2008 update, and the classification of the pathology of the lymphoepithelial ring of the pharynx [8, 33, 34]. Special attention was paid to the diagnosis of pathological conditions from the group of nasal obstructive disorders, in particular for HPT and AINS. The involvement of sinuses in the pathological process was diagnosed using the criteria of the European Position Paper on Rhinosinusitis and Nasal Polyps and the classification of the pathology of the lymphoepithelial ring of the pharynx [32].

### 2.3. Assessment of Nasal Respiratory Function

The respiratory function of the nose was assessed by the AARM method using the Rhino 31 computer rhinomanometer (Atmos, Germany) in accordance with the available guidelines [35]. The computer software allowed obtaining the parameters of the volume of the respiratory flow passing through the right and left half of the nose, total nasal airflow (TNAF), the resistance of the nasal structures of the right and left halves of the nose, and total nasal airway resistance (TNAR). The nose resistance was automatically calculated at a pressure of 75, 150, and 300 Pa/cm^3^/s. Below, only the data for the pressure of 150 Pa/cm^3^/s corresponding to the reference anthropometric data of [27] will be discussed. For all measurements, there was a strong correlation between the values of TNAF and TNAR,* R*^2^ = 0.998. Therefore, in the subsequent presentation of the results, only the values of TNAF will be given.

The study was carried out in the sitting position, one nostril of the patient was completely blocked with a special foam rubber roller, and the patient was asked to breathe calmly and uniformly through a silicone mask with the mouth closed. The measurement results were displayed in real time in the form of a rhinogram and after the measurement was completed, in the form of a diagram stored in the computer memory. Assessment of the degree of nasal obstruction was performed in accordance with the following TNAF indices: no nasal obstruction (> 800 cm^3^/s), mild nasal obstruction (500-800 cm^3^/s), moderate nasal obstruction (300-500 cm^3^/s), severe nasal obstruction (100-300 cm^3^/s), and complete nasal obstruction (<100 cm^3^/s) [10].

Clinical evaluation of AR symptoms was performed using the Total Nasal Symptom Score (TNSS) scale [36]; a quantitative assessment of the level of BA control was performed using the Asthma Control Questionnaire-5 (ACQ-5) questionnaire test. For ACQ-5 test values less than 0.75 points, the BA control level was considered complete, at ACQ-5 values from 0.75 to 1.5 points, the control level was assessed as partial, at values more than 1.5 points, a lack of control of asthma was declared [37].

*Spirographic studies *were performed using the MasterScreen Pneumo spirometer (Jaeger, Germany) in accordance with existing international guidelines [38]. The forced vital capacity (FVC), forced expiratory volume per second (FEV1), and the ratio FEV1/FVC were evaluated; the data were recorded both in absolute values of the indices and in relative units (in comparison with the relevant values determined, taking into account age, sex, body height, and ethnicity) [38].

### 2.4. Statistical Analysis

The statistical analysis was carried out using the Statgraphics Centurion v.9 software package. The data are presented in the form of Me [Q1; Q2], where Me is the median and [Q1; Q2] is 95% confidence interval. The differences between continuous variables of two groups were compared using the ANOVA criteria and Mann-Whitney U test. The differences between three groups were analyzed using multiple ANOVA criteria and the Kruskal-Wallis test. Correlations between the two sets were estimated using the Spearman determination coefficient. The values of p<0.05 were considered statistically significant.

## 3. Results

In all examined patients with asthma, AR was verified as persistent 86.4% (57/66) or intermittent 13.6% (9/66). This strong association of BA and AR is consistent with our previously published data, as well as with the data provided by Blaiss et al. [2, 39]. According to the severity of AR, the following distribution of patients was obtained: 12.1% (8/66) of children were diagnosed with AR of mild course, moderate AR was found in 81.8% (54/66), and severe AR was found in 6.1% (4/66) of the children. Exacerbation of AR was diagnosed in 48.5% (32/66) of the examined children and in 51.5% (34/66) there was a remission of AR. In addition, 69.7% (46/66) of children with asthma had concomitant NOD.

In the AARM evaluation of the respiratory function, the median TNAF value was 576.7 [519.6; 633.8] cm^3^/s. Differences between TNAF in boys (*n* = 40) of 565.8 [497.8; 633.9] cm^3^/s and in girls (*n* = 26) of 593.4 [487.3; 699.4] cm^3^/s were not detected,* p* = 0.64. In contrast, the groups of patients in different AR periods had significantly different TNAF values,* p* < 0.0001. Group A of patients in the remission of AR had the median TNAF values of 734.5.1 [687.8; 781.2] cm^3^/s which corresponded to a mild degree of nasal congestion. For Group B (exacerbation period of AR), these indicators were 409.0 [339.1; 478.9] cm^3^/s, which corresponds to the congestion of an average degree of severity.

**Figure 1:**
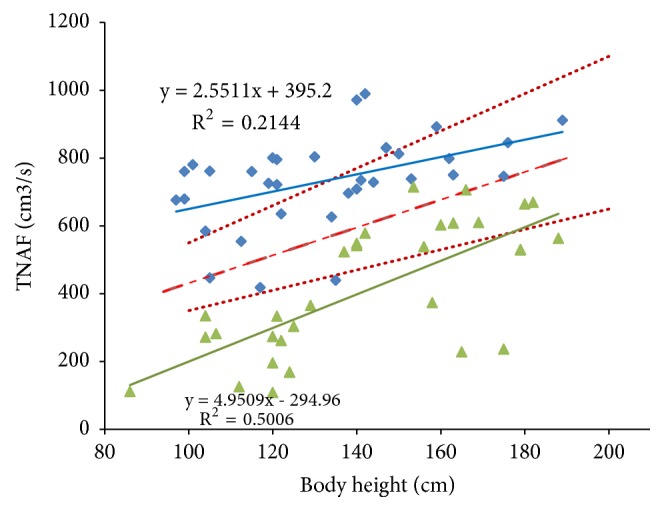
Measured values of TNAF for BA patients with AR remission (blue squares) and AR exacerbation (green triangles) depending on patients' body height. The linear regressions for groups A and B are shown by solid lines with corresponding regression expressions. Red dotted and dashed lines indicate the mean and reference intervals for healthy subjects (mean±2SD) taken from [27].

The measured TNAF values depending on their body height are shown in Figure 1. In this figure, the patients from Groups A and B of different AR severity are designated with different markers. The analysis shows that there is a statistically significant correlation between TNAF and body height both in the full cohort (*n*=66,* R*=0.299,* p*=0.0148) and in the patients with different AR period. In the period of exacerbation, the correlation between TNAF and body height is most pronounced (*n*=32,* R*=0.71,* p*<0.0001) whereas in the period of remission the correlation is significantly lower (*n*=34,* R*=0.46,* p*=0.0058). The linear regressions for patients of Groups A and B are shown in Figure 1 by solid lines.

One of the most important issues when discussing the AARM results in children is the question of the reference rhinomanometric values and their dependence on anthropometric parameters of patients [9, 13, 27, 28]. Earlier, Zapletal and Chalupovà [27] elaborated the reference TNAF values from the measurements of 192 healthy children and obtained the regression dependency between TNAF (in cm^3^/s) and body heights* h* (in cm) for mixed (boys+girls) population. Although this dependence was published in a logarithmic form, it has essentially linear character and we approximate it by the linear expression

(1) $$\begin{array}{c} \text{TNAF}=4.10483h+23.5735. \end{array}$$

The confidence interval of the reference TNAF values for healthy patients [27] is shown in Figure 1 with dashed and dotted lines in a form of (mean±2SD). As a whole, the measured TNAF values of patients with exacerbation of AR are lower than the reference values of healthy subjects although some of patients demonstrate the TNAF values comparable to the healthy children. The patients with the AR remission have remarkable higher TNAF values which are even higher than the reference values. In our opinion, this can be explained by the fact that all or most patients with BA usually receive vasoconstrictive drugs that provide a significant increase in the airflow.

It is also evident from Figure 1 that the dependence of TNAF on the body height is much stronger in the exacerbation period. Perhaps this is a reflection of the fact that the lower the body height, the smaller the size of the nasal cavity and the more the nasal flow is limited in the conditions of allergic inflammation, leading to swelling and inflammatory infiltration of the mucous membrane of the nasal cavity.

Strong dependence of measured TNAF values on the age and body height of patients makes it difficult to fulfill a direct comparison between different groups of patients assigned in our study because different groups contain different numbers of children with distinguished anthropometric characteristics. Thus, we need to elaborate a special index taking into account the age of patients. Given the statistically significant linear relationship between TNAF and the body height obtained in [27] (see (1)), we introduce the quantity of the Reduced Total Volumetric Airflow (RTNAF) which we define as

(2) $$\begin{array}{c} RTNAF=\frac{\left(TNAF\;–\;23.574\right)}{\left(4.1048h\right)}. \end{array}$$

**Figure 2:**
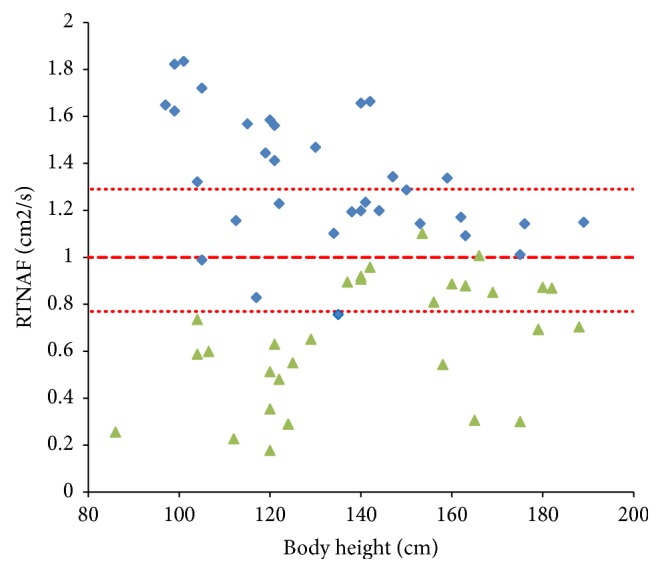
RTNAF values for BA patients of different height with AR remission (blue squares) and AR exacerbation (green triangles). Red dashed and dotted lines indicate the reference intervals for healthy subjects (mean±2SD).

Here, TNAF is a measured total nasal airflow in cm^3^/s,* h* is the body height in cm, and the measurement units of RTNAF are cm^2^/s. This quantity includes the body height, not the age of the children, since the nasal flow obviously depends on the size of the patient's nasal cavity, which is primarily related to the length of the child's body [9]. The convenience of the RTNAF index is that it equals 1 in the case of healthy subjects regardless of their age and height. This is demonstrated in Figure 2 where two groups of patients (A and B) are shown along with the 2SD intervals of healthy subjects [27]. More importantly, this index allows comparing the groups which combine the patients of different age and anthropometric characteristics. In the following, we will compare these groups using both TNAF and RTNAF values in order to estimate the possible influence of the mixing of patients of different age in the groups under investigation.

Similarly in TNAF, comparison of the RTNAF values between Groups A and B also shows the strong correlation with the AR phase: RTNAF group median value is 1.32 cm^2^/s in patients in the period of remission of AR and 0.69 cm^2^/s in the period of exacerbation,* p *< 0.0001.

Table 1 demonstrates comparison of the clinical indicators of nasal and bronchial patency (TNSS and FEV1), severity of BA (ACQ-5), and the rhinomanometric parameters (TNAF and RTNAF) during two periods of AR.

As is evident from the table, TNAF values correlate with subjective assessment of nasal symptoms made with TNSS score (*R* = –0.35 at* p* = 0.0054). The relationship between RTNAF is even more significant and amounted to* R* = –0.39 at* p* = 0.0015. This indicates a higher correlation of nasal symptoms with RTNAF in comparison with TNAF and may indicate certain advantages of using this value in assessing nasal respiratory function in children of childhood.

As is expected, the TNSS value is lower in patients in the period of AR remission compared to the period of AR exacerbation,* p* = 0.0004. It should also be noted that, in patients who have AR remission, there is a clear tendency toward higher FEV1 values and statistically significantly lower ACQ-5 values. This indicates that both bronchial permeability and clinical parameters of asthma control in children with AR are superior to those in children with acute AR. This is consistent with the concept of “single airway - a single disease” [40].

The statistically significant correlation between TNAF values and ACQ-5 scores was not found in the considered sampling,* p* = 0.098. However, a certain positive correlation was found between TNAF and FEV1 which is characterized by the determination coefficient of* R *= 0.34,* p *= 0.044. The correlation between RTNAF and the same parameters (ACQ-5 and FEV1) is more significant; the corresponding coefficients are* R* = –0.28,* p *= 0.025 for ACQ-5 and* R* = 0.37,* p *= 0.003. Thus, the nasal patency has significant relationship with clinically determined level of BA control in children if the anthropometric data of patients is taken into account, e.g., using the RTNAF index.

Tables 2 and 3 show the results of AARM measurements in the groups with different structure of comorbid pathologies in different AR periods. As is evident from the last column of tables, the groups of patients 1, 2, 3, and 4 of patients with different UAP comorbidities are significantly different in their nasal patency in the period of both AR remission and exacerbation. However, Fisher's test results shown in Tables 2 and 3 demonstrate only that the null hypothesis (Groups 1, 2, 3, and 4 are not distinguished) is wrong, and do not allow making detailed comparison between them. For the paired discrimination between the groups of patients with various UAP structures, Fisher's least significant difference (LSD) procedure for TNAF and RTNAF values was additionally carried out. This procedure discriminates the groups comparing the group means with the confidence limits of their variations. The results of such an analysis are shown in Tables 4 and 5. The groups with the mean differences that exceeded the confidence limits are attributed to being significantly different (within 95% confidence). These values are marked in tables with boldface. As is evident from the tables, Group 1 is distinguished from Groups 2 and 4 for both AR phases, whereas Groups 2, 3, and 4 are mutually distinguished only during the AR exacerbation. At the same time, discrimination based on the RTNAF values shows that Group 1 is always distinguished from Group 4 and from Group 2 in the exacerbation period. Among other groups, only Groups 2 and 3 are distinguished in exacerbation period. In our opinion, the different conclusions from the analysis of TNAF and RTNAF values are the consequence of inadequate accounting for anthropometric data when TNAF values are used. From this point of view, when TNAF and RTNAF data lead to different conclusions, we consider the analysis based on RTNAF values as more correct.

When analyzing TNAF in the period of remission of AR in children with various combinations of UAP, it was established that the highest nasal respiratory flow rates were in patients with isolated AR who do not have a combination with NOD. TNAF in patients with a combination of AR and AINS in this sample is slightly lower than in children with isolated AR, but the differences are not statistically significant (Table 4). The lowest absolute values of TNAF were found in children who had combinations AR+HPT and AR+HPT+AINS. The differences of these groups with the groups of isolated AR were statistically significant. Perhaps this is partly due to the fact that children with HPT were generally younger and had a lower body height than children without HPT. Age and height of children with HPT were 6.9 [5.7; 8.2] years and 124.6 [117.4; 131.9] cm, respectively. The age and body height of children without HPT were 10.8 [9.3; 12.3] years and 150.3 [141.3; 159.2] cm, respectively. The differences both in the age and in body height between these groups are statistically significant,* p* <0.00002.

The results of analysis of the RTNAF values in children in the period of AR remission demonstrate that the highest respiratory nasal patency is in children with isolated AR which is consistent with the results obtained in the analysis of TNAF. At the same time, a statistically significant decrease in RTNAF relative to the patients with isolated AR was found only in the group with AR+HPT+AINS. Differences in RTNAF values between Group 1 (isolated AR) and Group 2 (AR+HPT) were not established. The differences between Group 1 and Group 3 (AR+AINS) have a tendency character. This may indicate a relative compensation of nasal respiratory function in children in the period of AR remission who have a single variant of NOD only.

For the period of AR exacerbation, the TNAF values in all four groups were significantly lower than for the period of AR remission. The analysis of the RTNAF values demonstrates similar patterns. Namely, decrease in RTNAF values reflecting nasal patency reduction was observed in the exacerbation period compared with the period of remission. The changes relative to Group 1 are statistically significant for all groups except Group 3 (AR+AINS). This can be a consequence of the fact that Groups 2 and 4 have significantly decreased TNAF/RTNAF values which are not different from each other.

It should be noted that the lowest values of both TNAF and RTNAF in the period of acute AR were found in children with a combination of AR+HPT. A slightly lesser decrease in nasal respiratory function was found in children with a combination of AR+HPT+AINS. Thus, it seems that the most negative contribution to the formation of nasal congestion during the AR exacerbation is caused by adenoid vegetation; the influence of AINS on the indices of nasal patency in AR exacerbation is less pronounced.

## 4. Discussion

In the present work, we have studied nasal respiratory function in children with BA and AR using the AARM method taking into account the AR severity period and the absence or presence of two kinds of comorbid NODs. We have also evaluated the RTNAF index as an indirect indicator reflecting the nasal respiratory function, allowing eliminating effect of the patient height on the AARM measures.

As a whole, patients with asthma and nasal symptoms had a decrease in nasal respiratory function, which corresponded to an average degree of nasal obstruction. The median values of TNAF obtained in our study were 576.7 [519.6; 633.8] cm^3^/s. This is consistent with the data obtained in a study by Harmanci et al. [41], where the TNAF values in BA children were 554±79 cm^3^/s in the pollen season.

It was found that the nasal respiratory flow values, evaluated both by the direct measurement (TNAF) and using the relative RTNAF index, are significantly higher in children during AR remission than in children with acute AR. Although this fact is expected, there are some contradictions in the available publications about the significance of TNAF in children with BA. So, Yukselen et al. [20] observed the average degree of nasal obstruction in children with asthma with TNAF = 328±177 cm^3^/s. It should be noted that the causes of nasal obstruction were not elucidated in their study and the children's age was from 6 to 15 years. At the same time, Chen I. et al. [9] observed a mild degree of nasal obstruction with TNAF of 755.2 ± 337.8 cm^3^/s in children with BA from 5 to 18 years of age. In our study, the similar group of patients is characterized by the TNAF value of 734.5 [687.8; 781.2] cm^3^/s during the AR remission and 409.0 [339.1; 478.9] cm^3^/s during the AR exacerbation. Thus, our results demonstrate clear and statistically significant correlation with the severity period of comorbid pathology. Design of the study [20] excluded patients with anatomical deformities of the nasal cavity and, in the study [9], the state of the nasopharyngeal tonsil, the level of control of AD, and the period of AR were not accounted.

The differences in the nasal flow rate in children with BA, given in different sources, can be associated with several factors. First, the activity of allergic inflammation (remission or exacerbation of AR, as a rule, taking place in these patients) was not considered. Second, the anthropometric and age parameters of the child were not taken into account. Third, the structure of comorbid UAP, including NOD, that potentially can occur in these children was not accounted. This indicates the relevance of standardizing the evaluation of indicators of AARM in children, including patients with asthma, which has objective difficulties in comparison with the category of adults.

We have established that the studied indicators (direct measurement of the nasal flow of TNAF and estimation of reduced values of RTNAF) both in the period of remission of AR and in the period of acute AR have the highest values in children with isolated AR compared with children who have a combination of AR and NOD. In the period of AR remission, the most significant limitations of the nasal respiratory function were observed in children with the combination AR+HPT+AINS, as evidenced by both TNAF measurements and RTNAF index. In the AR exacerbation period, any NOD types as a whole have a negative impact on the nasal respiratory function in comparison with isolated AR, but the most pronounced decrease in nasal respiratory function is observed in children with HPT, as in the case of a combination of AR+HPT and with the combination of AR+HPT+AINS. This is evidenced by the analysis of both TNAF and RTNAF values. Thus, in children with asthma and nasal symptoms in the period of AR remission, significant deterioration in nasal respiratory function is observed mainly in children who have a combination of AR with HPT and AINS. In the period of exacerbation of AR, the most significant contribution to the disruption of the nasal respiratory function is made by HPT, both in the form of a combination of AR+HPT and in the form of AR+HPT+AINS.

We found single publications, mainly Zicari A.M. and coauthors, indicating a decrease in TNAF to 629.1 ± 146 cm^3^/s in children with HPT, which was significantly lower than in children who did not have HPT (age of patients from 6 to 12 years, patients did not have respiratory allergies) [42]. These data are consistent with the data obtained by us: during the remission phase of AR in children with HPT, TNAF values were 654. 8 [599.3;710. 3] cm^3^/s. Studies that reflect the effect of HPT on rhinomanometric parameters in children with asthma have not been found.

During the acute phase of AR, children with HPT had the lowest values of both TNAF and RTNAF, which indicates a significant negative effect of HPT on the respiratory function of the nose in children during acute AR. Similar patterns were obtained in patients who have a combination of AR+HPT+AINS (somewhat higher than in children with AR+HPT). In the period of exacerbation of AR, patients with HPT show the most significant decrease in nasal respiratory flow.

We have not established a significant influence of AINS (mainly in the form of deviations of the nasal septum) on the parameters of AARM in the period of remission of AR in children with asthma, both in the analysis of absolute TNAF values and in the analysis of the RTNAF index in comparison with children who had isolated AR. This, perhaps, may be due to the compensatory capabilities of one of the half of the nose, having a larger volume with AINS. When analyzing the values of the RTNAF index, it was established that, in the period of AR remission, the value of the nasal flow when leveling the effect of anthropometric parameters in children with HPT is statistically not distinguished from the nasal flow, of patients having isolated AR.

Analysis of TNAF in a group of children who had a combination of AR with AINS in the acute phase of AR showed a tendency to decrease nasal patency in patients with AINS, in comparison with children with isolated AR. In the period of exacerbation of AR, the absolute values of TNAF, but not RTNAF rates, are decreased as compared to the isolated AR. Thus, the effect of AINS on the nasal flow during the AR exacerbation period is not obvious.

The influence of AINS on the rhinomanometric parameters of the respiratory function of the nose in the children's population, and in particular in patients with asthma, is not adequately covered in the literature. We found mainly studies performed in adults as a criterion for assessing the respiratory function in surgical intervention for the correction of AINS [43]. In children, such studies are single, performed in patients who do not have asthma [44]. It should also be noted that Ahn et al. [45] established a high associative relationship of the AINS with asthma, in those cases where the AINS was accompanied by a syndrome of chronic nasal obstruction. The assessment of nasal obstruction in [45] was based on the results of the questionnaire and endoscopic examination of the nasal cavity. An objective assessment of the degree of nasal obstruction in patients with AINS and BA was not carried out in [45]. The AARM measurements can lead to more detailed interpretation of the AINS influence on the course of asthma. All this, undoubtedly, requires the continuation of studies on the effect of AINS in patients with BA and on the formation of nasal obstruction during the course of asthma.

## 5. Conclusions

Rhinomanometric studies in children with asthma and nasal symptoms show a clear dependence of the severity of nasal obstruction from the AR phase (remission or exacerbation). In addition, a negative effect on the state of nasal respiratory function of HPT, more pronounced with exacerbation of AR, was found, which should be taken into account when managing these patients. The effect of HPT on the decrease in nasal flow in the period of remission of AR is less significant than in the period of exacerbation. The effect of AINS on the nasal respiratory function of the nose is not found. Our results demonstrate that the interpretation of the AARM indices in children with asthma and nasal symptoms should take into account the entire spectrum of the existing pathology of UAP in this category of patients. At the same time, when analyzing the AARM parameters, it is important to take into account the influence of the patient's anthropometric indices on the parameters of the nasal respiratory flow.

## Figures and Tables

**Table 1:** Indices of the respiratory function of the nose according to clinical assessment of symptoms of AR (TNSS), asthma control (ACQ-5), spirometric indicators (FEV1), and AARM measurements in patients during different periods of AR.

Measured parameter	All patients, *n* = 66	Group A (AR remission, *n*=34)	Group B (AR exacerbation, *n*=32)	Statistics of difference between groups A and B
TNSS, scores	5.03 [4.41; 5.65]	3.97 [3.03; 4.90]	6.06 [5.39; 6.74]	*t* = –3.73 *p *= 0.0004 *W* = 719.5 *p* = 0.002
FEV1, % of req. val.	94.7 [90.5; 98.8]	99.0 [93.2; 104.9]	90.1 [84.5; 95.7]	*t* = 2.33 *p* = 0.026 *W* = 90.0 *p *= 0.039
ACQ-5, scores	0.60 [0.47; 0.74]	0.37 [0.25; 0.52]	0.83 [0.63; 1.02]	*t* = –3.73 *p *= 0.0004 *W* = 733.5 *p *= 0.0009
TNAF, см^3^/с	576.7 [519.6; 633.8]	734.5 [687.8; 781.2]	409.0 [339.1; 478.9]	*t* = 7.98 *p *< 0.0001 *W* = 76.0 *p *< 0.0001
RTNAF, см^2^/с	0.99 [0.89; 1.10]	1.33 [1.24; 1.43]	0.64 [0.55; 0.74]	*t* = 10.503 *p *< 0.0001 *W* = 27.0 *p *< 0.0001

**Table 2:** Measured TNAF values (cm^3^/s) in BA patients with different UAP structure in the periods of remission and exacerbation of AR.

AR periods	Group 1 (isolated АR)	Group 2 (AR+HPT)	Group 3 (AR+AINS)	Group 4 (AR+HPT+AINS)	Statistics between groups 1, 2, 3, 4
Both AR periods *n *= 66	771.0 [711.9; 830.0], *n*=20	456.1 [391.9; 520.0], *n*=17	609.6 [536.3; 682.9], *n*=13	435.3 [369.3; 501.4], n=16	*F*=12.75, *p*<0.0001 *KWT*=25.9, *p*<0.0001

Group A (AR remission) *n* = 34	811.0 [768.0; 853.9], *n*=15	654. 8 [599.3;710. 3], *n*=9	745.2 [670. 5;819.7], *n*=5	638.0 [563.5;712. 5], *n*=5	*F*=4.82, *p*=0.0074 *KWT*=12.22, *p*=0.007

Group B (AR exacerbation) *n* = 32	650.8 [567.1; 734.6], *n*=5	232.5 [166.3; 298.7], *n*=8	524.9 [458.7; 591.1], *n*=8	343. 2 [286.8; 399.7], *n*=11	F = 13.9, p < 0.0001 KWT = 18.2, p = 0.0004

Statistics between groups A and B	*F*=12.31, *p*=0.0025; *KWT* = 8.55, *p* = 0.0034	*F*=53.5, *p*<0.0001; *KWT* = 12.0, *p* = 0.0005	*F*=13.28, *p*=0.0039; *KWT* = 7.76, *p* = 0.005	*F*=10.71, *p*=0.0056; *KWT* = 6.5, *p* = 0.011	

**Table 3:** RTNAF values (см^2^/с) in BA patients with different UAP structure in the periods of remission and exacerbation of AR.

AR periods	Group 1 (isolated АR)	Group 2 (AR+HPT)	Group 3 (AR+AINS)	Group 4 (AR+HPT+AINS)	Statistics between groups 1, 2, 3, 4
Both AR periods *n *= 66	1.29 [1.17; 1.42], *n*=20	0.94 [0.81; 1.07], *n*=17	0.94 [0.78; 1.09], *n*=13	0.74 [0.60; 0.87], *n*=16	*F* = 6.49, *p *= 0.007 *KWT *= 15.46, *p* = 0.0015

Group A (AR remission) *n* = 34	1.42 [1.32; 1.52], *n*=15	1.36 [1.24; 1.48], *n*=9	1.22 [1.05; 1.39], *n*=5	1.12 [0.96; 1.29], *n*=5	*F* = 2.00, *p* = 0.14 *KWT* = 5.7, *p* = 0.12

Group B (AR exacerbation) *n* = 32	0.91 [0.77; 1.06], *n*=5	0.47 [0.36; 0.58], *n*=8	0.76 [0.65; .87], *n*=8	0.56 [0.46; 0.65], *n*=11	*F* = 5.57, *p* = 0.004 *KWT *= 11.05, *p* = 0.01

Statistics between groups A and B	*F* = 21.13, *p* = 0.002 *KWT *= 10.7, *p* = 0.001	*F* = 46.4, *p* < 0.0001 *KWT *= 12.0, *p* = 0.0005	*F* = 20.30, *p* = 0.0009 *KWT *= 8.59, *p* = 0.003	*F* = 14.22, *p* = 0.0021 *KWT *= 7.1, *p* = 0.008	

**Table 4:** Discrimination between different groups of BA patients in different AR periods on the basis of Fisher's least significant difference (LSD) procedure for TNAF values. The differences between groups are statistically significant (marked with boldface) if their Difference exceeds the Limit value.

Compared	AR remission		AR exacerbation	
groups	Difference	+/– Limit^*a*^	Difference	+/– Limit^*a*^
1-2	**156.2**	**99. 3**	**418.3**	**150.9**
1-3	65.8	121.6	125.9	150.9
1-4	**173.0**	**121.6**	**307.6**	**142.8**
2-3	–90.4	131.3	**–292.4**	**132.4**
2-4	16. 8	131.3	–110.7	123.1
3-4	107.2	148.9	**181.7**	**123.1**

^*a*^95% confidence limit of Fisher's least significant difference (LSD) procedure.

**Table 5:** Discrimination between different groups of BA patients in different AR periods on the basis of Fisher's least significant difference (LSD) procedure for RTNAF values. The differences between groups are statistically significant (marked with boldface) if their Difference exceeds the Limit value.

Compared	AR remission		AR exacerbation	
groups	Difference	+/– Limit^*a*^	Difference	+/– Limit^*a*^
1-2	0.061	0.216	**0.445**	**0.255**
1-3	0.200	0.266	0.156	0.255
1-4	**0.298**	**0.266**	**0.356**	**0.241**
2-3	0.139	0.289	**–0.289**	**0.223**
2-4	0.240	0.289	–0.090	0.208
3-4	0.098	0.328	0.199	0.208

^*a*^95% confidence limit of Fisher's least significant difference (LSD) procedure.

## Data Availability

The data used to support the findings of this study are available from the corresponding author upon request.
